# End-to-End Deep Learning Method for Detection of Invasive Parkinson’s Disease

**DOI:** 10.3390/diagnostics13061088

**Published:** 2023-03-13

**Authors:** Awais Mahmood, Muhammad Mehroz Khan, Muhammad Imran, Omar Alhajlah, Habib Dhahri, Tehmina Karamat

**Affiliations:** 1 College of Applied Computer Science, Almuzahmiyah Campus, King Saud University, Riyadh 11543, Saudi Arabia; 2 Department of Robotic, and Artificial Intelligence, Shaheed Zulifkar Ali Bhutto Institute of Science and Technology, Islamabad 44000, Pakistan; 3 Department of Software Engineering, Foundation University Islamabad, Islamabad 44000, Pakistan

**Keywords:** elderly care homes, Parkinson’s disease, prediction PD

## Abstract

Parkinson’s disease directly affects the nervous system are causes a change in voice, lower efficiency in daily routine tasks, failure of organs, and death. As an estimate, nearly ten million people are suffering from Parkinson’s disease worldwide, and this number is increasing day by day. The main cause of an increase in Parkinson’s disease patients is the unavailability of reliable procedures for diagnosing Parkinson’s disease. In the literature, we observed different methods for diagnosing Parkinson’s disease such as gait movement, voice signals, and handwriting tests. The detection of Parkinson’s disease is a difficult task because the important features that can help in detecting Parkinson’s disease are unknown. Our aim in this study is to extract those essential voice features which play a vital role in detecting Parkinson’s disease and develop a reliable model which can diagnose Parkinson’s disease at its early stages. Early diagnostic systems for the detection of Parkinson’s disease are needed to diagnose Parkinson’s disease early so that it can be controlled at the initial stages, but existing models have limitations that can lead to the misdiagnosing of the disease. Our proposed model can assist practitioners in continuously monitoring the Parkinson’s disease rating scale, known as the Total Unified Parkinson’s Disease Scale, which can help practitioners in treating their patients. The proposed model can detect Parkinson’s disease with an error of 0.10 RMSE, which is lower than that of existing models. The proposed model has the capability to extract vital voice features which can help detect Parkinson’s disease in its early stages.

## 1. Introduction

Parkinson’s disease (PD) is a progressive nervous system disorder that causes tremors, stiffness, or slowness in movement [1]. It is a disease of the brain that increases over time. Parkinson’s disease can cause many complications such as difficulty in thinking, depression or emotional changes, problems in swallowing, problems in eating or chewing, sleep disorders or restlessness, and problems in urination and constipation. Parkinson’s disease affects the daily routine movement of a person and it greatly affects automatic movements; Parkinson’s disease affects the ability to perform unconscious movements such as smiling or even blinking of eyes. In various cases, we have observed body parts impairment due to Parkinson’s disease.

The probability of developing Parkinson’s disease is higher in older adults. It is alarming that it affects 1% of the total population of older people [2]. It has affected over 10 million people worldwide. This disease is a severe threat to older adults, so the maximum chance of finding people with Parkinson’s disease is in elder care homes.

Parkinson’s disease detection can be difficult as some of its symptoms are like those exhibited in cold weather; for instance, shivering of the voice and movements. Therefore, there is a need to propose a technique which capable of extracting the important features which play a vital role in detecting Parkinson’s disease.

The critical detail of Parkinson’s disease is that it is a progressive disease, which means the disease progresses over time and if, somehow, we diagnose this disease in the early stages, it will be possible to eradicate it by using proper medication. The diagnosis of this disease is not as easy as it seems; it occurs mainly in older people, and in old age, there is a weakness that causes shivering. From the literature, it has been observed that different techniques and methods are used for the classification of Parkinson’s disease.

Several reasons can take part in the development of Parkinson’s disease in any patient. For example, genes, environmental change, age, and the presence of Lewy bodies which are substances found in the brain. Age is a very important factor in Parkinson’s disease, as most patients with Parkinson’s disease are people with an age greater than 50. Another major reason is genetics. There is a strong probability of developing Parkinson’s disease if it is in the genes. As Parkinson’s disease occurs in mostly elderly people, it is very difficult to distinguish Parkinson’s disease symptoms from other kinds of shivering that usually occur in elderly people. The most suitable method to detect this disease is using voice signals. Parkinson’s disease is a progressive disease of the brain. The Unified Parkinson’s Disease Rating Score (UPDRS) is used to find out the patient’s current situation. This scale helps in finding whether a patient is suffering from Parkinson’s disease or not.

There are two parameters in UPDRS that can be used by data scientists to find out the possibility of Parkinson’s disease in a patient by using different techniques.

Parkinson’s disease prediction in the real environment is more difficult as there are many challenges in the process, for example, in the case of detecting Parkinson’s disease using voice signals, there is a factor of noise in the signal which can heavily affect the results. Another challenge in the same process is the mixing of voice signals, as the voice signal of a single person can be mixed up with that of the another person and existing models will give the results on the input signal without differentiating it from the signal of the other person. Such challenges have made the prediction of Parkinson’s disease in patients in a real environment very difficult. Accuracy is another important factor in the validation of a model for the prediction of Parkinson’s disease.

As Parkinson’s disease is increasing day by day and it occurs mostly in elderly people, our aim is to develop a model that will receive real-time data from elderly homes. A home with voice recorders can help us to obtain the necessary voice signals and then apply signal preprocessing filters, and, by using clustering techniques, we can divide these signals for patient recognition, and, within these clusters, we can apply our model to find out the is the patients has symptoms of Parkinson’s disease.

Parkinson’s disease has affected more than 10 million people around the world, in other words, 40 out of 100,000 demonstrate the effects of this disease. The motivation behind this research is to provide safety to our senior citizens living in elder homes from this harmful disease. Several existing models can detect Parkinson’s disease but have limitations that can lead to the misdiagnosing of the disease. This is the reason that the use of such models in old age homes could be problematic.

Early diagnosis of Parkinson’s disease is essential for the treatment of Parkinson’s disease patients and existing literature is unable to devise a reliable method that can detect Parkinson’s disease in its early stages. There are multiple factors to detect the presence of disease in a patient and existing models have limitations in contending with the multivariate characteristic of the dataset which leads to a high error rate in diagnosing Parkinson’s disease [3,4,5,6,7].

In this research, we developed a model for the detection of Parkinson’s disease through the voice signal. We extracted the features from the audio signal and developed a model based on acoustic deep learning for the early identification of disease for early- treatment of patients. This study uses different preprocessing techniques on the voice signals before the extraction of the various features from the given dataset. After feature extraction, the PCA was utilized to reduce the feature dimensions. Finally, acoustic deep learning is applied to the reduced feature vector to produce better results. The basic objective of this research is to reduce the error rate in the diagnosis of Parkinson’s disease using voice features.

The related work is discussed in the second section. It is followed by Section 3, which is the proposed methodology. Then we explore the data graphically, preprocess data, and fit the data in our model to obtain results in Section 4. Finally, we conclude the paper by summarizing it in Section 5.

## 2. Literature

Rastegar et al. [3] presented a model that detects Parkinson’s disease by using random forest on cytokine data. Cytokine molecular data reveal important information regarding the clinical phenotypes. Cytokine molecules help in signaling the immune system. The authors applied random forest for the classification of a dataset with records of 360 different persons that was provided by the Michael J Fox Foundation. The tree-like structure of the random forest helped detect Parkinson’s disease by using entropy and information on the provided data. The proposed model was evaluated by finding out the toot mean square error (RMSE), which was 0.1123 for the Hoehn and Yahr scale and 0.1193 for the Unified Parkinson’s Disease Rating Scale part three (UPDRS III) [3].

Nilashi et al. [4], presented a remote tracking model that consisted of a clustering approach to predict Parkinson’s disease by using voice data. In this model, the UCI dataset of 5875 instances was utilized to assess the performance of the proposed model. The proposed study suggested the use of cluster methodology to predict the diseases successfully. SOM (self-organizing map) was used to cluster the data based on similarity. The clusters grouped by SOM were used by artificial neural networks for classification. These similar clusters were used by artificial neural networks for classification. In the proposed model, these clusters are sent for learning in the next step which consists of a deep neural network. They evaluated the proposed model by root mean square error score and the model was able to score 0.537 on test data. This model tracks the Unified Parkinson’s Disease Rating Scale score of a patient on a regular basis to check the progression of Parkinson’s disease.

A regression-based model for the detection of the disease was proposed by [8] where a neural network and decision tree were utilized to that detect Parkinson’s disease. Saloni et al. [9] proposed a similar approach [4] to detect Parkinson’s disease on the Unified Parkinson’s Disease Scale by using voice data. The proposed technique uses a support vector machine for the identification of Parkinson’s disease on the Unified Parkinson’s Disease Scale. In the proposed model, data consist of 197 instances collected from the UCI inventory. Initial preprocessing was conducted, where basic stats were applied to check the null values, mean, and median of the data to check the skewness in data. These checks helped in the improvement of error, as the presence of null values or the presence of skewness in data can badly affect the model generalization. The performance of a learning model highly depends on the cleanliness of the data. The pre-processed or cleaned data can significantly improve the performance of a learning model. Null data or null values were removed by imputing the mean of the data and skewness in the data was improved by using standardizing data techniques. These preprocessed data were sent for regression analysis by using a support vector machine. The presented model was evaluated based on the root mean square error and 0.24 root mean square error produced by the model.

Chen et al. [10] presented a deep learning model for the effective prediction of Parkinson’s disease using the voice data of a patient. In the proposed model, the number of neurons and the selection of activation function play important role in correctly classifying data and obtaining the R2 score of around 96% on the training data.

Nooritawati et al. [11] presented a different approach to predicting Parkinson’s disease in a patient by using the gait movement of patients. The proposed model mainly focuses on three important characteristics of gait movement: spatiotemporal, kinematic, and kinetic. In the proposed model, features from the gait movement were extracted, such as spatiotemporal, kinematic, and kinetic, and then converted into numerical form. These uncleaned data were preprocessed by using intra-group normalization and inter-group normalization. These preprocessing techniques helped in extracting spatiotemporal, kinematic, and kinetic features of the gait movement. The extracted data were then sent to the artificial neural network and support vector machine for classification purposes. The presented model of the artificial neural network was able to yield an accuracy of 98%. Linear support vector machine was used for the classification of healthy and Parkinson’s disease patients [12]. A low-cost diagnostic model for the early diagnosis of Parkinson’s disease was proposed by [13]. Deep multivariate vocal data analysis (DMVDA) applies different deep learning classifiers [14] for the detection of disease. Maachi et al. [15] presented a 1D-Convnet deep learning convolutional neural network for the prediction of Parkinson’s disease. The concept of transfer learning with convolution neural network was applied by [16,17,18,19]. Deep neural networks, support vector machines, convolution neural networks, and ensemble deep neural networks explored by [20,21,22,23,24] for the prediction of Parkinson’s disease.

Ashour et al. [25], proposed a monitoring procedure for the detection of Parkinson’s disease using a recurrent neural network.

Gunduz et al. [26], proposed two convolution neural networks for the detection of Parkinson’s disease using voice signals. In the proposed study, two convolution neural networks of nine layers were introduced and the performance of was evaluated using root mean square error (RMSE). In the first convolution neural network of nine layers, feature sets are combined before being given to the network, while, in the second architecture, feature sets are given parallel to convolutional layers and these are merged after the second convolution layer. These architectures are trained by using voice data and classification of Parkinson’s disease conducted by a support vector machine. The deep neural network-based model by [27] uses the voice of patients for the classification of Parkinson’s disease. Dai et al. [28] use U-net architecture for better diagnosis of Parkinson’s disease. In the proposed model, many preprocessing techniques are applied such as histogram equalization, gray level transformation, improved wavelet soft-threshold de-noising, and image enhancement on positron emission tomography (PET) images of the patient after preprocessing the data sent to a convolutional neural network model based on U-net architecture. Multi-layer perceptron-based model is proposed by [29] that can detect multiple neurological disorders including Parkinson’s disease. Haq et al. [30] proposed a model for the detection of Parkinson’s disease using the voices of patients. The proposed model consists of pre-processing for dealing with missing values and scaling problems, an L1-norm SVM algorithm for feature extraction, k-fold cross-validation for hyper-parameters, and a support vector classifier. The model was evaluated based on a confusion matrix, and the best accuracy of 99% was achieved on selected nine features by using the L1-norm SVM algorithm. Prince et al. [31] proposed a model for the monitoring and detection of Parkinson’s disease by using wearable sensors on the body of the patient. The wearable sensors can help in monitoring the activities or vitals of a person under examination without a physical inspection of the person. Relief-F for feature reduction was explored by [32] to improve the classification accuracy on large datasets.

In the literature, we observed lots of models and frameworks based on machine learning algorithms and deep learning algorithms to detect Parkinson’s disease by using the voice, gait, and handwriting of patients. These three techniques play an important role in the detection of Parkinson’s disease as voice, gait movement, and handwriting show significant change when a person experiences Parkinson’s disease. A person can experience a significant change in voice, gait movement, and handwriting because Parkinson’s disease affects the whole body through mental health. The detection of Parkinson’s disease through voice signals was mainly focused on in the literature as the highest accuracy and benchmark datasets were available publicly. The lowest error record in the literature was 0.112 by using random forest [3]. The study with the lowest RMSE in the literature still does not deal with the multivariate characteristic of the dataset and gives no detail about the importance of features that can help in detecting Parkinson’s disease.

## 3. Proposed Model

In the literature, we observed the absence of useful techniques that can help detect Parkinson’s disease with less error rate. In the proposed model, we have introduced useful and significant techniques for detecting Parkinson’s disease. After acquiring the data, it is necessary to process data before introducing them into the learning model. The raw data usually contain anomalies, consume a large amount of power, and occupy a large amount of memory and the cost of using raw data is relatively higher than that of processed data. The raw data consume lots of time and resources for processing and can badly affect the model’s performance in terms of generalizability and results. The raw data can contain noise or unnecessary information, leading to the wrong interpretation of the working of the model and results. Therefore, a learning model must learn from clean data.

The performance and generalizability of the learning model can be enhanced by using the processed data. The generalizability of the model can be defined as the ability of the model to predict values for unseen data, and a model needs to have good generalizability. The presence of outliers and unscaled data are the main reasons for the low performance of the model. The pre-processing techniques help make a learning model efficient and give a clear idea about the dataset.

The generalizability of the model depends upon the preprocessing of the data because the model should be able to produce results on unseen data, and if the training data have anomalies, then it would be difficult for a model to generalize on unseen data. The reliability of the model is of great importance in the production environment, and the reliability of the model can be determined by measuring the performance of the model on unseen data.

The presence of high dimensions is also the main reason for observing a significantly high error rate in the learning model, the presence of high-dimensionality data or many features makes it difficult for learning models to predict perfectly.

The characteristics of the dataset include a multivariate characteristic, which means there is a correlation among the features of the data. The presence of correlation in the dataset indicates that features are proportional to each other, so the increase or decrease value of one feature can affect the other feature. The performance of the learning model can be affected by the correlated features. To deal with the correlation among the features of the dataset, various techniques help in removing the correlated features. For further identification of the correlated features of the dataset, a heatmap is usually used to display the correlation among the features of the dataset. Once the correlated features are identified, one of them can be removed from the dataset so that it does not have any significant effect on the performance and results of the learning model. The high dimensional correlated data can badly affect the results of the learning model. The high correlation among the dataset features allows learning models to estimate the relationship between dependent features and independent features.

**Figure 1:**
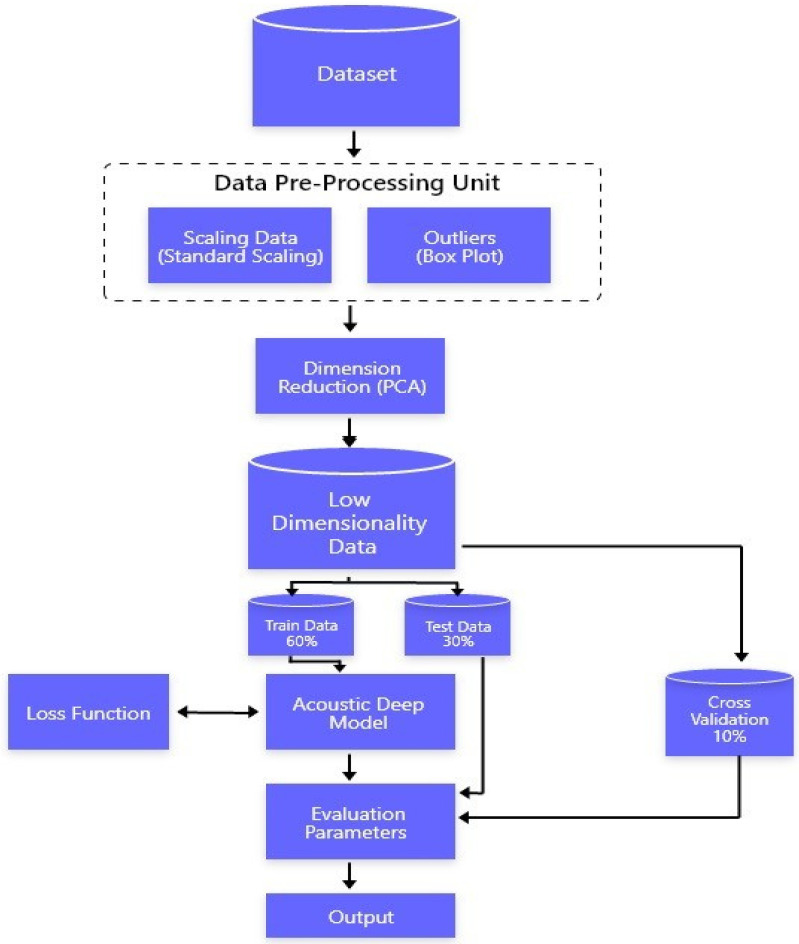
Proposed model.

Voice data have several different features. These features are always correlated with each other, so determining the effect of each feature and the importance of each feature that can help detect Parkinson’s disease. The proposed model is presented in Figure 1.

This study aims to conduct research on the detection of Parkinson’s disease by using voice signals. During the literature study, the use of machine learning and deep learning classifiers was closely monitored and critically analyzed. In the proposed model, the multivariate dataset is preprocessed by taking care of outliers and standardizing the dataset. We used Standard Scalar to standardize our data [6,8]. We used box plot techniques to find outliers [5], which is an efficient technique to detect outliers from the data. Once the data were wholly clean, we dealt with multi-variate characteristics of the data. The principal component analysis does dimension reductions by finding the correlation between the different features of the data. Once we were able to remove the multivariate property of the data, it was ready for the learning phase; the resulting dataset was transferred to an acoustic deep learning neural network where backpropagation help the model to reduce the error by minimizing the loss function [9,12]. The acoustic deep learning model was trained on 60% of the training data and tested on 30% of the testing dataset while the rest of the 10% was used for cross-validation. This network learns trends from data and gives results on a scale of total UPDRS. The total UPDRS score of the patient will help us in detecting the current situation of the patient. The Total UPDRS, which is a rating scale from 0–108, will help us detect progressive Parkinson’s disease. After training, a model is evaluated based on the root mean square error. During the literature review, we observed that deep learning models played a significant role in improving accuracy and reducing errors. A model with multiple layers with the right values of weight and biases can generalize data with minimum error. During the literature review, it was observed that deep neural networks can learn trends much better than other machine learning models.

The data point at a particular instance *i* can be represented below.

(1) $$D=\left\{\left(X_{i,}\;y_{i}\right)\right\},\;1\leq i\;\leq N$$

where $X_{i,}$ is the vector of input at a particular point *i,* while $y_{i}$ is the output of the corresponding input. The output function can be described below.

(2) $$f\left(X_{i}\right)=W^{T}\emptyset \left(X_{i}\right)+b$$

where *W* is the weight, *b* is the bias, and ∅(X) maps the input vector to dimensional features. *W* and *b* can be calculated by solving optimization equation.

(3) $$Min\frac{1}{2}\;{\left|\left|W\right|\right|}^{2}+C\;\overset{N}{\underset{i=1}{\sum }}\left(\varepsilon_{i}+\varepsilon_{i}^{*}\right)$$

subject to:

(4) $$y^{i}-W^{T}\left(\emptyset \left(x\right)\right)-b\leq \epsilon +\varepsilon_{i}\;W^{T}\left(\emptyset \left(x\right)\right)+b-y_{i}\;\leq \epsilon +\varepsilon_{i}^{*}\;\varepsilon_{i},\varepsilon_{i}^{*}\geq 0$$

where *C* refers to the predefined positive trade-off between the generalizability and model simplicity. To measure the cost of error, $\varepsilon_{i},\varepsilon_{i}^{*}$ are used.

Before implementation of acoustic deep neural network, principal component analysis (PCA) was used for the dimensionality reduction. The covariance between the two variables or the features gives us information regarding the trends in the data. For instance, if value of one variable is increasing due to which the value of other variable is increasing too, it will mean that there is positive covariance between them. In other words, if the covariance of two variables is positive it means that these two variables are directly proportional to each other; if the covariance between the two variables is negative, it will mean that they are indirectly proportional to each other. The covariance between the two variables can found by the following equation.

(5) $$COV\left(X,Y\right)=\frac{1}{n-1}\overset{n}{\underset{i=1}{\sum }}\left(X_{i}-x\right)(Y_{i}-y)$$

In the above equation, “*X_i_*” is the data value *x*, “*Y_i_*” is the data value *y*, “*x*” is the mean of the X feature, “*y*” is the mean of the Y feature, and “*n*” is the total number of values.

To implement the acoustic deep neural network, we proposed building a model of neurons and different layers of neurons, which contains weights and biases that we adjust according to the loss function.

**Figure 2:**
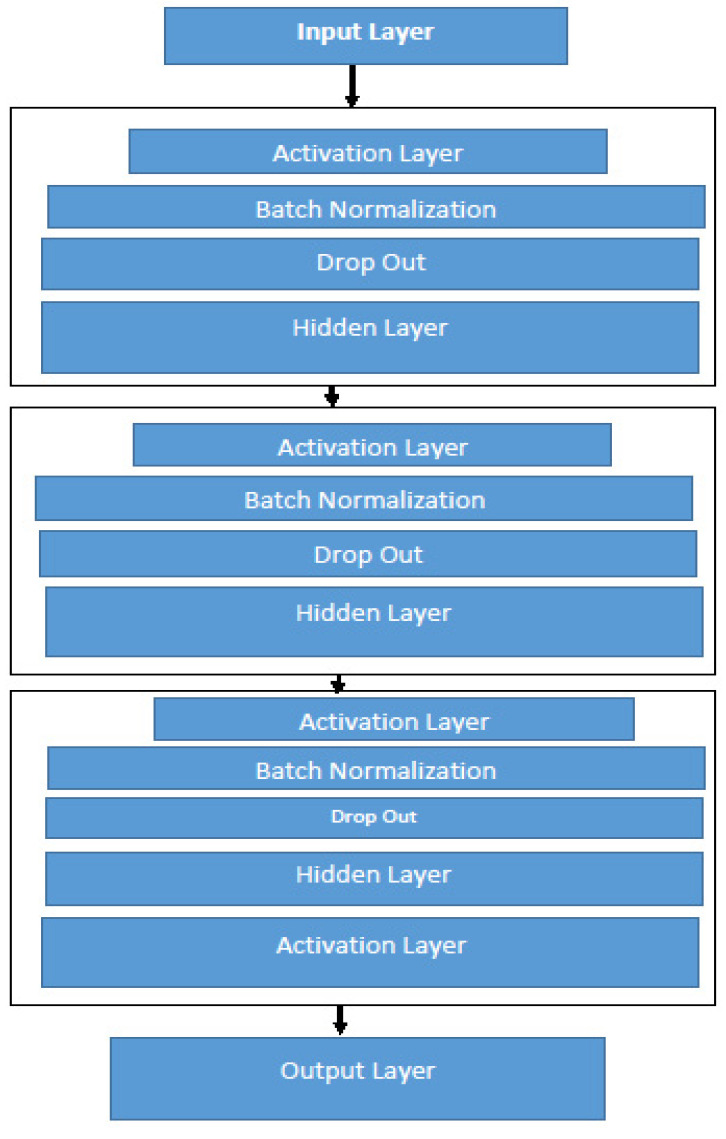
Proposed deep learning architecture.

The model shown below in Figure 2 is a conceptual model of the acoustic deep learning model that will detect Parkinson’s disease. The proposed model diagram explained *b* comprises feature selection and an acoustic deep learning model. This learning model has the capability of detecting Parkinson’s disease with great accuracy and precision. This acoustic deep learning model consists of a complex network of neurons and weights connected to them. The large numbers of weights can learn the pattern and trends in the data and detect the right value of each record of the data.

## 4. Experimentation

The dataset used in our study can be retrieved from the UCI Machine Learning Datasets repository [33]. This dataset contains 16 attributes including Shimmer: APQ11; Shimmer (dB); Shimmer: NR, DDA, PPE, NHR, DFA, RPDE; Shimmer: APQ5; Jitter: DDP, Jitter (Abs); Jitter: PPQ5; and Jitter: (%). This multivariate dataset is based on voice recordings collected from 42 patients over 6 months. Voices of patients and healthy people are recorded and target variables such as Total UPDRS and motor UPDRS were measured by clinical examination, Each subject has almost 200 recordings during the six-month trial [33]. These multivariate data consist of 22 features, including the motor UDRS and combined Parkinson’s disease scale. This dataset is recognized as the benchmark dataset used for the identification of this disease using voice data. In the literature, this dataset was widely used [33]. These data are suitable for the regression as it displays the data which depict the progressive value for the Total Unified Disease Scale rating for a patient [4,9,10,33].

After the UCI retrieval of the dataset for the disease from the repository, the very first step performed on the dataset was data exploratory analysis. In this step, the basic stats were derived from the dataset, which majorly includes finding the mean of each of the data features. The median, mode, variance, and standard deviation were also calculated in this step. We calculated the median to determine the center value of the dataset. Similarly, for the datasets, the statistical values of variance along with the standard deviation were calculated.

**Figure 3:**
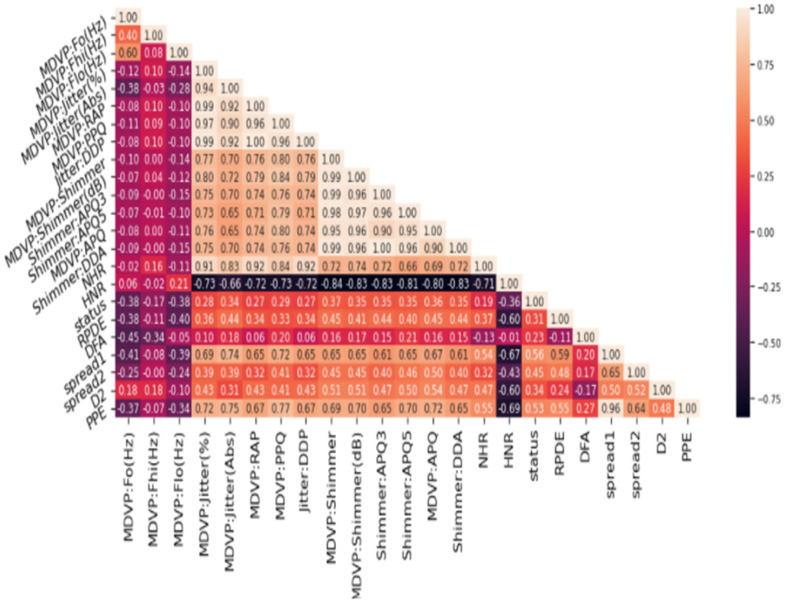
Heatmap.

Variance and standard deviation helped us understand the degree of spread of the data; this helped measure the dispersion in the dataset. The exploratory data analysis gave us a good idea about the data. The exploratory data analysis helped in identifying the obvious errors and interesting relationships between the features of the dataset. For instance, the following heatmap showed (in Figure 3) the multivariate characteristic of the data features of the dataset. This heat map showed the features are correlated to each other in one or the other. We observed a correlation between the different attributes of the dataset. Correlation can be termed as the mutual relationship between two objects. If we correlate our datasets then, with the increase in value of one column, we will observe an increase in value of other columns, this is known as a positive correlation. If an increase in values of one column causes a decrease in another column, it is referred to as a negative correlation.

We observed that Parkinson’s disease has affected a specific age group during the exploratory data analysis. As this disease is considered a continual neural disorder, it develops over time and the dataset clearly shows that Parkinson’s disease has affected people of age between 50 and 90. A density graph is shown below, showing the density of Parkinson’s disease among a specific age group.

**Figure 4:**
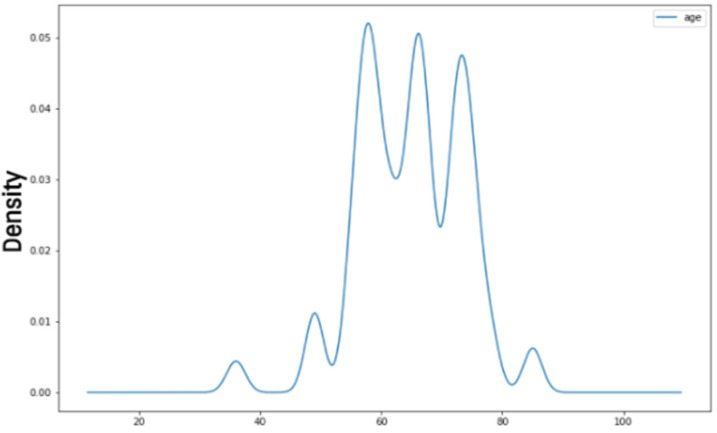
Age Density graphs.

The Figure 4 shows that regular monitoring of Parkinson’s disease in older people is necessary as it can develop in later stages of life. So, a technique that can detect Parkinson’s disease on a scale will be the most adequate in this regard. The results deduced in the analysis process for exploration set bases for applying the preprocessing methods.

During the preprocessing step, we applied two major techniques and solved two major problems. One was removing outliers from the features of the dataset as outliers can greatly affect the model’s generalizability and negatively affect the performance of the model. Outliers are the data points that differ significantly from the other data points. The presence of a single outlier can greatly change the value of the standard deviation and can change the whole spread; therefore, it is necessary to deal with the outliers before pushing data to the model for training purposes. The indication of outliers was conducted by using the box plots on the features of the dataset. The box plots divide the whole feature of a dataset into quarters and the density of each quarter can help us build an understanding of the data. The points that lie away from the minimum and maximum boundaries of the box plot can be considered outliers; thus, these outliers are then removed from our dataset. After the removal of outliers, it is important to scale the data. The machine learning algorithm we used to calculate distance and scaling helps reduce the chances for a feature to dominate in distance measurements. The performance of the machine learning model can be negatively impacted by this. For that purpose, we used scaling methods to reduce the chance for a feature to dominate the calculation of distance. Scaling can be conducted in different ways, such as via the min–max scaler, mean scaler, standard scaler, etc. Scaling data will specify each data point of the feature of the dataset to a specific range. We used standard scaling for scaling the data into a specific range.

There are 16 dimensions of the UCI Parkinson’s disease dataset, and the heatmap below shows a correlation between different features of the datasets in our exploratory analysis of the data. After scaling the features of the dataset, we reduce the dimensions of the dataset. For this, we identified the required elements of dataset which are key for better performance and generalizability of the model. For this purpose, we used the principal element analysis method.

**Figure 5:**
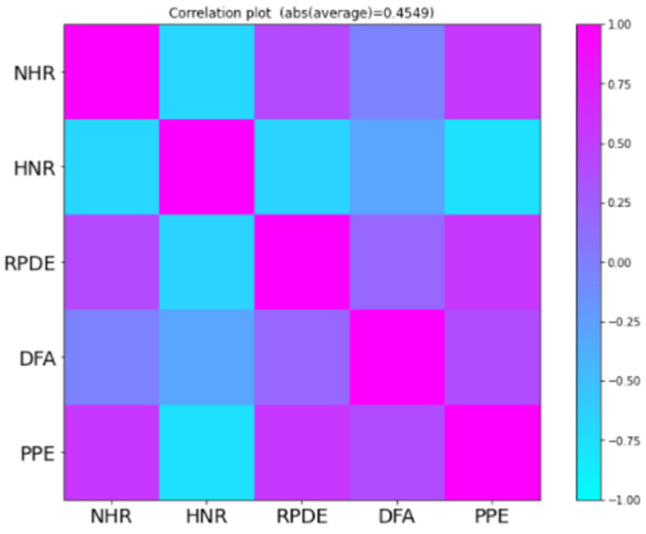
Correlation between selected features.

We used principal component analysis to determine the required components or features of the dataset that are vital for the better performance and generalizability of the model. Figure 5 shows the correlation between selected features.

After applying the principal component analysis, training required splitting datasets, testing, and validation sets. The division followed a ratio of the testing set (30%), training set (60%), and validation set (10%). The deep learning model we created for identifying this disease by using audio samples of the patients trained on 60% of the total dataset, which we named training data. This model learned the trends, internal behaviour, and internal relationship among the different features of the dataset and the relationship with the expected output. The proposed model used the backpropagation technique with activation functions, which helped learn the behaviour better.

The root means square error and r-squared score were the metrics which were applied to measure the performance of the potential model. The error value between the predicted and real data points was the basis to measure the proposed model. The distance between these gives an exact performance of the model.

The Total Unified Parkinson’s Disease Scale ranges from 0 to 108, in which 0 shows the absence of Parkinson’s disease or complete health of the patient with no disability, and 108 represents the worst disability. The actual point and predicted point distance are calculated with these evaluation parameters which are then called the Total Unified Parkinson’s Disease scale. The result achieved by the proposed framework is presented in Table 1.

To achieve better results than previous models used by different experiments, we used metrics for evaluation which proved to be providing better results [3,4,9]. The R-squared of 86% shows that 86% of the variance in the dependent variable is explained by the model. The existing models in the literature used root mean square error and R-squared to evaluate the capability of model. In the literature, some of the models were evaluated on only one parameter, while some of the models were evaluated based on two parameters. Therefore, we also used the same metrics to compare the proposed framework. The result of the proposed method is compared with the existing method as shown in Table 2.

**Figure 6:**
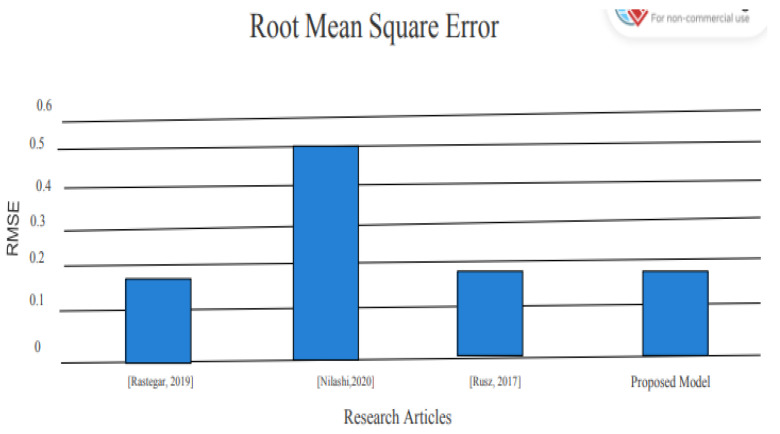
RMSE comparison [3,4,29].

After implementing the proposed model, the results are significant and show a better advancement in detecting Parkinson’s disease by using voice data. The RMSE, which shows the error in detecting the presence of Parkinson’s disease in patients, was only 0.10, where the previous models Nilashi et al. [4] and Saloni et al. [9] reported RMSE of 0.537 and 0.24, respectively, which is less than the RMSE reported by the proposed model. Table 2 clearly shows that R-squared reported by Nilashi et al. [4] is 78%; however, the proposed model reported R-squared value is 85%. These numbers of performance parameters clearly indicate that proposed model performance is better than the existing models for the detection of Parkinson’s disease. To express the result in graphical form, the same result comparison shown in Figure 6.

The performance comparison of the proposed model with existing literature based on RMSE is shown in Figure 6.

**Figure 7:**
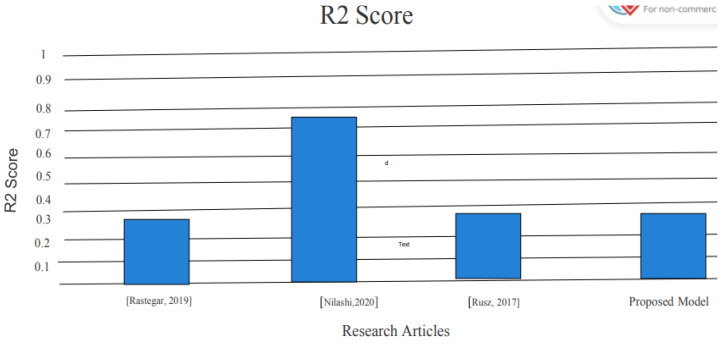
R-squared score comparison [3,4,29].

Moreover, we have also reported an R-squared error which explains the extent to which data are close to our predicted regression line. The R-squared of 86% shows that the model explains 86% of the variance in the dependent variable. The R-squared of the proposed model was compared with the existing models and the results show that the proposed model has a significant impact; its results are better than the results of the existing studies. A comparison based on the R-squared score is shown in Figure 7.

This comparison shown in Figure 7 between the results of the existing model and the proposed model indicates that the performance of the proposed model is better than the existing models. The R-squared of the proposed model is 86%, which shows that our model is much more reliable than the existing models because our model can explain the variance in the targeted variable up to 86% due to the independent variables.

## 5. Conclusions

Early diagnosis of Parkinson’s disease is necessary for the treatment of the patient. If a patient receives an early diagnosis of Parkinson’s disease, then there is a greater chance that the patient will recover from the situation by using the proper treatment and medication. In today’s world, computer sciences have made a lot of advancements, especially in the fields of machine learning and deep learning, which have revolutionized the medical field. The use of state-of-the-art machine learning and deep learning algorithms has made the lives of people easy. These algorithms have played a vital role in classifying different diseases, monitoring patient health, and early prediction of diseases. The detection of Parkinson’s disease in its early stages is vital for the treatment. In our study, we have gone through the extensive literature and observed the limitations in the detection of Parkinson’s disease by using the voice data of the patients. After going through the extensive literature and finding out the limitations, based on these limitations, we proposed a model that will assist in the elimination of the limitations that already exist in the literature. The proposed model can detect the presence of Parkinson’s disease in its early stages with great accuracy. With our proposed model, we are confident that it will bring a revolutionary change in this field and assist practitioners in detecting the presence of Parkinson’s disease in a patient in the early stages of the disease. The proposed model has the capability of diagnosing Parkinson’s disease early. To extend this study, we will explore the other deep learning models presented in various studies [34,35,36,37,38].

## Figures and Tables

**Table 1:** Results of the proposed model with RMSE and R-Squared.

	RMSE	R-Squared
Proposed Model	0.10	86%

**Table 2:** Proposed method results comparison with existing methods.

Reference	RMSE	R-Squared
Ahmadi et al [3]	0.112	-
Nilashi et al. [4]	0.537	78%
Saloni et al. [9]	0.24	-
Proposed Model	0.10	86%

## Data Availability

The data used in this study already publically available at UCI machine learning web portal as mentioned in the experiments section.
